# Treatment Failure Outcomes for Emergency Department Patients with Skin and Soft Tissue Infections

**DOI:** 10.5811/westjem.2015.7.26213

**Published:** 2015-10-20

**Authors:** Larissa S. May, Mark Zocchi, Catherine Zatorski, Jeanne A. Jordan, Richard E. Rothman, Chelsea E. Ware, Samantha Eells, Loren Miller

**Affiliations:** * University of California, Davis, Department of Emergency Medicine, Davis, California; † The George Washington University, Office for Clinical Practice Innovation, Washington, District of Columbia; ‡ The George Washington University, Department of Emergency Medicine, Washington, District of Columbia; § The George Washington University, Department of Epidemiology and Biostatistics, Washington, District of Columbia; ¶ Johns Hopkins University, Department of Emergency Medicine, Baltimore, Maryland; || Los Angeles BioMedical Research Center at Harbor - UCLA Medical Center, Torrance, California

## Abstract

**Introduction:**

Skin and soft tissue infections (SSTIs) are commonly evaluated in the emergency department (ED). Our objectives were to identify predictors of SSTI treatment failure within one week post-discharge in patients with cutaneous abscesses, as well as to identify predictors of recurrence within three months in that proportion of participants.

**Methods:**

This was a sub-analysis of a parent study, conducted at two EDs, evaluating a new, nucleic acid amplification test (NAAT) for *Staphylococcus aureus* in ED patients. Patients ≥18 years receiving incision and drainage (I&D) were eligible. Patient-reported outcome data on improvement of fever, swelling, erythema, drainage, and pain were collected using a structured abstraction form at one week, one month, and three months post ED visit.

**Results:**

We enrolled 272 participants (20 from a feasibility study and 252 in this trial), of which 198 (72.8%) completed one-week follow up. Twenty-seven additional one-week outcomes were obtained through medical record review rather than by the one-week follow-up phone call. One hundred ninety-three (73%) patients completed either the one- or three-month follow up. Most patients recovered from their initial infection within one week, with 10.2% of patients reporting one-week treatment failure. The odds of treatment failure were 66% lower for patients who received antibiotics following I&D at their initial visit. Overall SSTI recurrence rate was 28.0% (95% CI [21.6%–34.4%]) and associated with contact with someone infected with methicillin resistant *S. aureus* (MRSA), previous SSTI history, or clinician use of wound packing.

**Conclusion:**

Treatment failure was reduced by antibiotic use, whereas SSTI recurrence was associated with prior contact, SSTI, or use of packing.

## INTRODUCTION

Skin and soft tissue infections (SSTIs) are common reasons patients seek medical care in the emergency department (ED).^1^ Between 2006 and 2010, there were 34.8 million outpatient visits for SSTIs, one-third of which were seen in the ED.^2^ Patients with cutaneous abscesses are more likely to be younger in age, of racial and ethnic minorities, and of low-income status.^3^ Although there are many risk factors associated with SSTI acquisition, these infections commonly affect otherwise healthy individuals.^4^

Patients with SSTIs are subject to the potential of both treatment failure and recurrence. Recurrences occur in 30–70% of persons following an initial SSTI, with rates greater than 50% in certain populations.^5^–^8^ Given the high rate of recurrence, SSTIs represent a significant healthcare burden to U.S. EDs, specifically in terms of increased cost, morbidity, and mortality. However, in spite of the high incidence of SSTIs in ED settings, very little is known about factors associated with treatment failure and recurrence in these patients.

Our primary objectives included the following: identify predictors of SSTI treatment failure in patients with a cutaneous abscess within one week of their initial ED visit, identify the proportion of ED patients with cutaneous abscess who develop recurrence within three months, and identify predictors of recurrent infections.

## METHODS

Data for this investigation were taken from a larger parent clinical trial of adults 18 years of age and older who were treated for a cutaneous abscess with incision and drainage (I&D) that will be reported in a separate manuscript (under review). This study, registered on clinicaltrials.gov (# NCT01523899) was conducted in two urban academic EDs from January 2011 through April 2014. The parent study was a randomized controlled trial comparing antibiotic selection in patients being screened using a new, FDA-cleared Xpert *Staphylococcus aureus* SSTI nucleic acid amplification test (NAAT) (Cepheid, Sunnyvale, CA) during their ED visit compared to standard-of-care testing. This study also included data from an additional 20 participants enrolled in a feasibility/pilot study that was run to ensure all data forms were usable and that the study could be conducted at both ED locations. Here we evaluated the factors associated with treatment failure and recurrence within that study population.^9^

Study personnel, stationed in the ED at both sites during daytime hours (generally 9 AM to 8 PM), consecutively screened potentially eligible patients for inclusion and exclusion criteria. Inclusion criteria included the following: 18 years of age and older, capable of providing written informed consent, complained of symptoms consistent with a possible abscess (e.g. abscess, SSTI, wound, ulcer, insect bite), and were receiving I&D for their abscess. Wound, nares, and inguinal site specimens were obtained and cultured for *S. aureus* during the enrollment visit. We excluded patients if they had received treatment for the same abscess within 14 days (including I&D), had taken systemic antibiotic therapy within 14 days, those with surgical site or post-procedure infections, or those whose abscesses did not yield purulent material for testing.

Potentially eligible patients were identified by research assistant screening the ED electronic tracking board for patient chief complaints and/or direct ED clinician referral. Patients who met criteria for enrollment were approached for written informed consent and collection of demographic, clinical, and diagnostic testing data. The institutional review boards at both ED sites approved this study.

A structured data abstraction form was completed by trained research staff during the ED visit from patients who provided written consent. Data collected included the following: demographic information (age, gender, race, comorbidities, insurance type); epidemiologic factors and exposures (antibiotic use in past six months, hospitalization in past year, living situation, number of household contacts including children under 18); abscess characteristics (size, location, presence of systemic symptoms), and choice and duration of antimicrobial therapy. All follow-up data were collected through phone calls via a structured survey created prior to study initiation. Follow-up telephone calls were conducted at 2–7 days, one month, and three months post-discharge and brief structured data abstraction forms were used. Patient-reported data at the one-week follow up included questions regarding improvement in erythema, swelling, pain, fever, and drainage. SSTI recurrence at one or three months was described by the reappearance of one or more symptoms consistent with a cutaneous abscess including swelling, erythema, pain and/or fever, reported during the one- and/or three-month follow-up telephone calls.

Patients who did not respond to the one-week follow-up phone call and did not have a record of returning to the ED for their two-day follow-up wound check were excluded from analysis. We did not include patients in recurrence analysis if they did not respond to both the one-month and three-month follow-up phone call.

### Wound Cultures

At clinical site A, wound culturing was performed as standard of care using direct plating per Clinical and Laboratory Standards Institute (CLSI) standards in the hospital microbiology laboratory. At clinical site B, wound swabs were stored at 4°C and shipped weekly on cold packs to Cepheid’s laboratory in Sunnyvale, CA, weekly and underwent both direct plating and broth enrichment culturing, per CLSI standards.

### Nasal and Inguinal Colonization Cultures

Nasal and inguinal swabs were immediately stored at 4°C and shipped weekly on cold packs to the Cepheid laboratory in Sunnyvale, CA, for broth enrichment culturing. Cefoxitin and oxacillin susceptibility was confirmed via disk diffusion using Mueller Hinton agar (Cat. #R01620, Remel, Lenexa, KS) as described by the CLSI.

### Data Analysis

Our primary outcomes included the following: a) failure to improve within one week following initial treatment and b) patient reported SSTI recurrence at one and/or three months of treatment of the index (enrollment) SSTI. We defined treatment failure as no change in or increased pain, swelling, erythema, drainage of the current abscess, or new or persistent fever greater than 100.4°F. SSTI recurrence was defined as presence of a new abscess (characterized by swelling, pain, redness or drainage) at the same or different location at least two weeks after resolution of the initial abscess. Operationally, these outcomes were determined by chart abstraction at the ED two-day follow-up visit (through ED provider documentation of patient’s reported symptom improvement) or by patient self-report data collected during the follow-up phone call interview. Due to the large number of patients who missed at least one of the follow-up visits, patients were only considered lost to follow-up (LTFU) if they missed both the one- and three-month follow up. We used descriptive statistics (mean, frequency) to describe the demographics, clinical features, wound and colonization culture results of the study population compared to participants LTFU. Chi-square or Fisher’s exact tests, as appropriate, were used for categorical variables and T-tests for continuous variables. Statistical significance was considered at the alpha=0.05 level.

To identify characteristics that were independently associated with treatment failure and infection recurrence, variables that were significant at a level of p<0.10 in the bivariate analyses were fitted in a logistic regression model using a backwards stepwise selection process. Models were examined for goodness of fit using the Hosmer-Lemeshow statistic. We conducted all analyses using Stata v. 13.1 (College Station, TX).

## RESULTS

We enrolled 272 participants in the study (20 from a feasibility study prior to study initiation and 252 patients enrolled in this trial). One hundred ninety-eight (72.8%) participants completed the one-week follow up, with one-week outcomes for an additional 27 participants obtained through medical record review. One hundred fifty-six (57%) participants completed the one-month follow up and 136 (50%) completed the three-month follow up for a total of 193 participants (71%) that completed either the one-month or three-month follow up. Completed follow up was defined as completing either the one- or three-month follow-up phone call. The overall LTFU rate was 29.0%. We compared participant demographics, clinical features, and wound culture results between those that completed follow-up (n=193) and those LTFU (n=79).

We found that the participants LTFU were more likely to be homeless or treated by providers with less experience than those who were not LTFU. The mean age of participants was 36.3 years of age. Forty-two percent of participants had Medicaid insurance. Majority of participants (62%) reported a history of SSTI within the past 12 months. Most abscesses were less than 5 cm in diameter (93%). Demographic, clinical, and treatment characteristics of the study population and those LTFU are shown in Table 1.

The measured one-week treatment failure rate was 10.2% (23/225). Unadjusted odds of treatment failure were reduced in patients prescribed antibiotics and increased in patients treated by a resident physician compared to a physician assistant. Other factors, such as whether the *S. aureus* cultured was methicillin susceptible strains (MSSA) or methicillin resistant *S. aureus* (MRSA) or demographic characteristics, were not significantly associated with treatment failure at one week. After adjusting for prescription and abscess location, treatment by a resident was no longer significantly associated with higher odds of a negative outcome (Table 3).

The one- and three-month SSTI recurrence proportion was 22.4% (35/156) and 19.9% (27/136), respectively, among participants who were successfully contacted. Combined, SSTI recurrence occurred in 28.0% (CI [21.6%–34.4%]) of the 193 patients contacted at one or three months.

In the bivariate analysis, variables associated with SSTI recurrence included the following: previous contact with someone infected with MRSA (per patient self-report), prior history of SSTI within the past 12 months, and clinician use of wound packing. After adding additional variables with significance of p<0.10 into the model (Table 2, Table 4), predictors that were found to increase odds of recurrence were as follows: previous contact with someone infected with MRSA, clinician use of wound packing, prior history of SSTI in the past two and 3–6 months (compared to none). Patients with one MRSA-positive colonization site (compared to no MRSA sites) were found to have reduced odds of SSTI recurrence. Prior history of SSTI in the past 7–12 months, and comorbid conditions were not found to be associated with SSTI recurrence (Table 2, Table 4).

## DISCUSSION

To our knowledge, our study is the first in the literature assessing rates of and factors associated with SSTI treatment failure and recurrence among ED patients. We found most patients with cutaneous abscesses recovered from their initial infection within one week, with only 10.2% of patients who completed follow up reporting treatment failure at one week. The odds of treatment failure was 66% lower for those patients who received antibiotics after I&D at their initial visit, while patients with buttock abscesses were more likely to have treatment failure, possibly due to the difficulty of draining these abscesses.

We reported high recurrence rates (28% within three months) of cutaneous abscess amongst ED patients, consistent with what is described in the literature from other settings.^5^–^8^ Of those patients who completed both the one- and three-month follow-up visits, 34% experienced a recurrence. This is likely an underestimate given that patients LTFU were more likely to be homeless, a population previously identified at high risk for SSTIs. We also found that a self-reported history of SSTI within the past six months and prior contact with someone infected with MRSA were significantly associated with recurrence. Published data reveal that the vast majority of purulent SSTIs are caused by *S. aureus,* with greater than 50% caused by MRSA*.* In the United States, the predominant community-associated MRSA (CA-MRSA) clone, USA300 MRSA, has been associated with an increasing incidence of CA-SSTI, which typically manifest as cutaneous abscesses.^10^,^11^ Contrary to previous studies,^10^ we found no difference in SSTI recurrence between patients with MRSA wound infections compared to those who did not have MRSA infection. Surprisingly, having a single site of MRSA colonization was, in fact, associated with decreased odds of recurrence. It is possible multiple sites of colonization may be associated with higher odds or that colonization may be transient in these patients.

While we hypothesized colonization by either MSSA or MRSA would be associated with recurrence, we did not find this to be the case. Several investigations in non-ED settings have found a relationship between MRSA nasal carriage and subsequent SSTIs and recurrence;^12^,^13^ however, this relationship has not been consistently observed.^14^,^15^ The lack of an observed association may be related to our relatively small sample size; however, it is also possible that it could be due to the transient nature of colonization, which may have been undetected during the ED visit. It is also plausible that in ambulatory settings, colonization is not associated with recurrence, as has been suggested by others.^11^ The lack of a relationship between *S. aureus* colonization and SSTI in the outpatient setting is supported by interventional studies in which decolonization does not result in decreased infection rates.^16^

A recent multicenter double-blind, randomized clinical trial compared the use of trimethoprim-sulfamethoxazole (TMP-SMX) and placebo on uncomplicated I&D procedures and recurrence outcomes.^17^ Follow ups were completed at two, seven, and 30 days following initial presentation. The study indicates that treatment with TMP-SMX does not reduce treatment failure but may decrease the recurrence of subsequent lesions.^17^ Another trial done in pediatric patients showed no difference in treatment failure when using antibiotics or placebos, suggesting that antibiotics are not needed for SSTI resolution.^18^ However, even though the vast majority of our outpatients had good clinical outcome, we did find that antibiotic therapy was associated with an improved outcome at seven days, suggesting a potential role for antibiotic treatment in patients with uncomplicated abscesses. Another recent clinical trial comparing placebo to TMP-SMX treatment in patients with uncomplicated cutaneous abscess found better outcomes in the latter group following I&D and are less likely to require hospitalization or have SSTI recurrence.^19^ From a treatment perspective, antibiotic therapy was not associated with decreased recurrence risk in our study, but the practice of wound packing was associated with increased odds of recurrence. The reasons for this latter observation is unclear; however, other studies have shown that this practice may not improve outcomes and can lead to increased pain.^20^

The factors that fuel recurrent MSSA or MRSA SSTIs are not well understood. Data suggest poor hygienic practices are associated with an increased likelihood of MRSA infection.^21^–^23^ Others have found minimal association between poor hygienic practices in patients with CA-MRSA or CA-MSSA infections and uninfected controls, although the scope of the question was limited to sharing towels and using antimicrobial soap.^24^ While there are mounting data on the role of behavioral factors in increased MRSA infection risk, our understanding of the relative impact compared to colonization and other factors is limited.

Consistent with our findings that there was no association between a specific pathogen and recurrence, a recent investigation demonstrated that 51% of 330 index patients, and 13% of household contacts suffer recurrent infection within six months of treatment of the index patient for a *S. aureus* skin infection.^21^ Recurrent infections in that study were not associated with an initial infection caused by MRSA, MSSA, USA300 MRSA, or with having a CA-*S. aureus* infection, consistent with our findings of a lack of association of pathogen with outcomes.^10^,^25^ While that study found an association with recent hospitalization, cephalexin use, diabetes mellitus, and recent skin infection, Miller et al also show an association with household *S. aureus* or MRSA fomite contamination.^21^ This potential important factor was not assessed in this study. Although there is evidence that *S. aureus* contamination in households is common,^26^–^28^ and that *S. aureus* can persist on fomites for months,^29^ the relationship between fomite contamination and infection risk is unclear.^11^

## LIMITATIONS

There were several important limitations to the study. First, while we attempted to enroll consecutive patients, patients were likely missed during the hours where research assistants were unavailable. Secondly, the study was conducted at two urban EDs, located in the same geographic region (Mid-Atlantic), which may not represent SSTI epidemiology nationwide. Finally, we may not have had sufficient power to assess all potential factors that might be associated with recurrent SSTI. We also had significant loss to follow up in our study population (29%). While there were no major significant differences other than homelessness between participants lost to follow up and those who completed follow up, it is possible that the recurrence rate might be higher amongst homeless patients, given the potential role of close contact and poor hygiene in MRSA transmission. In addition, the small sample size and low number of recurrences limited the number of predictors assessed and the study may not have been adequately powered to detect small differences. This may have been confounding in that patients who did not receive packing may have had less concerning abscesses that were less likely to recur. Another limitation is the potential for bias related to the original randomized control trial.

## CONCLUSION

In summary, to our knowledge this study was the first to describe factors associated with both clinical outcomes and recurrence of cutaneous abscess in the ED setting. We found treatment failure occurred in only 10.2% of our population who completed follow up, while recurrence occurred in approximately one-third of patients within three months. Treatment failure, but not recurrence, was associated with antibiotic use, suggesting antibiotics may play a significant role in clinical management despite previous evidence that most uncomplicated abscesses do not require them. Predictors of recurrence included prior SSTI history and close contact with someone infected with a SSTI.

Interestingly, packing was associated with increased recurrence, warranting further investigation of this practice. While antibiotic use was associated with a slight improvement of short-term outcomes, we did not find any association with recurrence rates, and thus, there was insufficient evidence to change the recommended practice of limited antibiotic therapy to patients with complicated SSTI following I&D. Also, we did not find that the presence of MRSA or MSSA was more closely associated with recurrence. Despite great concern by ED providers regarding MRSA, there is, to date, insufficient evidence that cutaneous abscesses caused by MRSA have worse outcomes than those caused by other infectious etiologies.

## Figures and Tables

**Table 1:** Demographic and clinical features of the participants.

Demographics	All participants, % (n=272)	Participants lost to follow-up, % (n=79)	Remaining participants, % (n=193)
Age, mean (SD)	36.3 (13.8)	36.7 (12.4)	36.1 (14.3)
Female	53.7	46.8	56.5
Race			
Black	69.9	72.2	68.9
White	13.2	15.2	12.4
Other	2.9	5.1	2.1
Missing	14.0	7.6	16.6
Insurance			
Private	38.6	32.9	40.9
Medicaid	41.9	49.4	38.9
Medicare	7.7	6.3	8.3
Other	0.4	0.0	0.5
Self-pay/uninsured	9.2	10.1	8.8
Missing	2.2	1.3	2.6
Any comorbidity	33.8	29.1	35.8
Comorbidities, type			
Diabetes	12.9	13.9	12.4
HIV/immunocompromised	6.6	6.3	6.7
Multiple	7.0	5.1	7.8
Other	7.4	3.8	8.8
Prior history of SSTI	61.8	53.2	65.3
Prior history, timing			
No prior history	25.3	31.8	22.8
Past 2 months	30.5	27.3	31.7
Past 3–6 months	14.2	10.6	15.6
Past 7–12 months	24.5	27.3	23.4
Unknown	5.6	3.0	6.6
Prior hospitalization	21.0	17.7	22.3
Household size			
2–4 in household	67.4	64.1	68.8
5 or more in household	16.9	16.7	16.9
Live alone	13.1	11.5	13.8
Homeless*	2.6	7.7	0.5
Children in household	42.3	43.0	42.0
Recent antibiotic use	37.1	30.4	39.9
Contact w/someone w/SSTI	18.1	13.9	19.8

Clinical features	All participants, % (n=272)	Participants lost to follow-up, % (n=79)	Remaining participants, % (n=193)
Abscess location			
Axilla	24.6	20.3	26.4
Buttock	21.0	21.5	20.7
Extremities	17.3	27.8	13.0
Face	11.4	11.4	11.4
Perineum	9.2	5.1	10.9
Trunk	16.5	13.9	17.6
Multiple abscesses	12.5	11.4	13.0
Abscess diameter			
<1cm	8.8	5.1	10.4
1–2cm	39.0	39.2	38.9
3–5cm	44.9	49.4	43.0
>5cm	7.0	6.3	7.3
Missing	0.4	0.0	0.5
Erythema size			
<2cm	51.8	45.6	54.4
3–5cm	30.5	32.9	29.5
>5cm	16.2	20.3	14.5
Missing size	1.5	1.3	1.6
Prescribed antibiotics	75.4	76.0	75.1
Prescriptions			
Beta lactams	6.3	3.8	7.3
Clindamycin	41.9	48.1	39.4
TMP-SMX	15.8	11.4	17.6
TMP-SMX and beta lactams	9.9	10.1	9.8
Other	1.5	2.5	1.0
None prescribed	24.6	24.1	24.9
Packing used	83.1	83.5	82.9
Irrigation & debridement used	98.5	98.7	98.4
Provider type			
PA	55.2	49.4	57.5
Attending	27.6	35.4	24.4
Resident	17.3	15.2	18.1
Provider experience**†			
<10	14.1	20.3	11.6
10–50	26.9	31.7	24.9
>50	59.0	48.1	63.5
Received test (vs control)	53.7	51.9	54.4

Pathogen Characteristics	All participants, % (n=272)	Participants lost to follow-up, % (n=79)	Remaining participants, % (n=193)
Wound culture result			
MRSA	28.3	30.4	27.5
MSSA	18.0	16.5	18.7
Other	47.4	46.8	47.7
No culture/no growth/missing	6.3	6.3	6.2
Any *S. aureus* colonization	47.8	49.4	47.2
*S. aureus* colonization sites			
None	52.2	50.6	52.8
One	22.8	26.6	21.2
Two	25.0	22.8	25.9
Any MRSA colonization	25.0	24.1	25.4
MRSA sites			
None	75.0	76.0	74.6
One	14.0	13.9	14.0
Two	11.0	10.1	11.4
One week follow-up			
Negative outcome	10.2	11.4	9.7

*HIV,* human immunodeficiency virus; *SSTI,* skin and soft tissue infections

* p=0.02.

*TMP-SMX,* trimethoprim-sulfamethoxazole; *PA*, physician assistant

** p=0.047.

† Number of prior incision and drainage procedures.

*MRSA,* methicillin-resistant *Staphylococcus aureus*; *MSSA,* methicillin-susceptible *Staphylococcus aureus*

**Table 2:** Bivariate analysis of risk factors for recurrence of skin and soft tissue infection and negative one-week outcome.

	Recurrence (n=193)			Negative 1 week outcome (n=225)		
	
Demographic variables	OR	CI	P	OR	CI	P
Age	0.98	0.95–1.01	0.12	1.01	0.99–1.05	0.30
Gender, % female	1.62	0.84–3.10	0.15	1.02	0.43–2.44	0.96
Race						
Black	1	Ref		1	Ref	
White	0.43	0.14–1.35	0.15	2.03	0.68–6.07	0.20
Other	2.17	0.30–15.9	0.45	1.68	0.19–14.67	0.64
Insurance						
Medicaid	1	Ref		1	Ref	
Private	0.87	0.44–1.75	0.70	1.79	0.72–4.47	0.21
Medicare	0.32	0.07–1.5	0.16	1	(empty)	
Self-pay/uninsured	0.94	0.30–2.98	0.92	1.56	0.30–7.95	0.60
Any comorbidities	0.77	0.39–1.50	0.44	1.08	0.42–2.78	0.86
Comorbidities						
None	1	Ref		1	Ref	
Diabetes	1.18	0.46–2.99	0.734	1.96	0.65–5.86	0.23
HIV/immunocompromised	1.05	0.30–3.61	0.944	1	(empty)	
Multiple	0.86	0.26–2.86	0.799	1.12	0.13–9.58	0.91
Other	0.15	0.02–1.15	0.068	0.75	0.09–6.15	0.79
Prior history of SSTI	4.24	1.86–9.65	<0.001	1.13	0.46–2.79	0.79
Prior history, when						
No prior history	1	Ref		1	Ref	
Unknown	1.54	0.44–5.37	0.50	0.3	0.06–1.62	0.17
Past 2 months	4.68*	1.58–13.90	<0.01	0.98	0.31–3.14	0.97
Past 3–6 months	4.84*	1.43–16.40	0.01	0.55	0.11–2.84	0.47
Past 7–12 months	1.98	0.60–6.57	0.26	0.94	0.29–3.00	0.91
Prior hospitalization (Y/N)	0.73	0.33–1.6	0.43	0.48	0.14–1.69	0.25
Household size						
Live alone	1	Ref		1	Ref	
2–4 in household	1.48	0.55–3.97	0.43	0.46	0.14–1.58	0.22
5 or more in household	0.77	0.22–2.75	0.69	1.68	0.44–6.48	0.45
Homeless	1	(empty)		1.00	0.09–10.66	1.00
Children in household	1.04	0.55–1.96	0.91	1.40	0.59–3.32	0.45
Recent antibiotic use	1.30	0.69–2.45	0.42	1.16	0.48–2.81	0.74
* Contact with someone with MRSA/boils	2.24	1.07–4.70	0.03	0.65	0.18–2.29	0.50

	Recurrence (n=193)			Negative 1 week outcome (n=225)		
	
Clinical features	OR	CI	P	OR	CI	P
Abscess location						
Axilla	1	Ref		1	Ref	
Buttock	0.64	0.26–1.57	0.33	3.28	0.80–13.52	0.10
Extremities	0.53	0.18–1.56	0.25	1.98	0.42–9.40	0.39
Face	0.37	0.11–1.27	0.12	0.79	0.08–8.00	0.84
Perineum	1.04	0.36–2.96	0.95	0.79	0.08–8.00	0.84
Trunk	0.36	0.13–1.03	0.06	3.57	0.86– 4.76	0.08
Multiple sites	1.25	0.50–3.09	0.63	2.06	0.70–6.06	0.19
Abscess diameter						
<1cm	1	Ref		1	Ref	
1–2cm	0.95	0.30–2.97	0.93	2.82	0.34–23.26	0.33
3–5cm	1.22	0.40–3.73	0.73	2.04	0.25–16.92	0.51
>5cm	3.00	0.70–12.88	0.14	1.27	0.07–21.97	0.87
Erythema size						
<2cm	1	Ref		1	Ref	
3–5cm	1.06	0.52–2.16	0.87	0.93	0.33–2.61	0.89
>5cm	0.68	0.25–1.85	0.45	1.53	0.50–4.68	0.46
Prescribed antibiotics	0.91	0.45–1.84	0.79	0.34	0.14–0.82	0.02
Prescriptions						
Clindamycin	1	Ref		1	Ref	
Beta lactams	1.63	0.51–5.20	0.41	1.02	0.11–9.10	0.98
TMP-SMX	0.46	0.17–1.27	0.14	2.79	0.72–10.73	0.14
TMP-SMX and Beta lactams	0.58	0.17–1.93	0.37	0.70	0.08–6.09	0.74
Use of packing	3.30	1.01–9.88	0.03	4.30	0.56–32.99	0.16
Use of irrigation and debridement	0.77	0.07–8.71	0.84	1	(empty)	
Provider type						
PA	1	Ref		1	Ref	
Attending	0.52	0.23–1.16	0.11	1.86	0.70 – 4.89	0.21
Resident	0.40	0.15–1.04	0.06	1.62	0.48 – 5.47	0.44
Provider experience*						
<10	1	Ref		1	Ref	
10–50	0.53	0.18–1.61	0.26	0.55	0.17–1.80	0.33
>50	0.66	0.26–1.73	0.40	0.42	0.14–1.26	0.12

	Recurrence (n=193)			Negative 1 week outcome (n=225)		
	
	OR	CI	P	OR	CI	P
Pathogen parameters						
Wound culture result						
MRSA	1	Ref		1	Ref	
MSSA	1.27	0.51–3.16	0.61	0.83	0.23–3.02	0.78
Other	0.95	0.10–2.59	0.88	0.91	0.33–2.48	0.86
*S. aureus* colonization	0.95	0.51–1.79	0.88	1.07	0.45–2.54	0.88
*S. aureus* colonization sites						
None	1	Ref		1	Ref	
One	0.81	0.35–1.87	0.62	0.73	0.22–2.40	0.60
Two	1.08	0.51–2.27	0.84	1.40	0.53–3.69	0.50
MRSA colonization	0.91	0.44–1.88	0.79	1.02	0.38–2.72	0.97
MRSA sites						
None	1	Ref		1	Ref	
One	0.31	0.09–1.10	0.07	0.55	0.12–2.51	0.44
Two	2.09	0.84–5.22	0.11	1.76	0.54–5.77	0.35

*HIV,* human immunodeficiency virus; *SSTI,* skin and soft tissue infections; *MRSA*, methicillin-resistant *Staphylococcus aureus*

*TMP-SMX,* trimethoprim-sulfamethoxazole; *PA*, physician assistant

* Number of prior incision and drainage procedures.

*MRSA,* methicillin-resistant *Staphylococcus aureus*; *MSSA,* methicillin-susceptible *Staphylococcus aureus*

**Table 3:** Adjusted odds ratio of negative one-week outcome (n=225).

	OR	CI	P
Provider type			
PA	1	Ref	
Attending	2.23	0.78–6.39	0.13
Resident	1.23	0.34–4.46	0.75
Antibiotics prescribed	0.33	0.13–0.87	0.03
Abscess Location			
Axilla	1	Ref	
Buttock	4.36	1.00–19.06	0.05
Extremities	2.82	0.55–14.56	0.22
Face	0.71	0.07–7.55	0.78
Perineum	0.89	0.08–9.33	0.92
Trunk	3.89	0.90–16.75	0.07

*PA,* physician assistant; *CI*, confidence interval; *OR*, odds ratio

**Table 4:** Adjusted Odds Ratio of Recurrence (n=193).

	OR	CI	P
Contact w/someone w/SSTI	2.87	1.19–6.93	0.02
Use of packing	4.64	1.39–15.46	0.01
Prior history of SSTI	4.25	1.79–10.12	<0.01
MRSA Sites			
None	1	Ref	
One	0.24	0.06–0.91	0.04
Two	1.67	0.59–4.71	0.33
Comorbidities			
None	1	Ref	
Diabetes	1.00	0.35–2.84	1.00
HIV/immunocompromised	0.54	0.14–2.11	0.38
Multiple	0.62	0.16–2.37	0.49
Other	0.17	0.02–1.45	0.11

*SSTI,* skin and soft tissue infections; *MRSA,* methicillin-resistant *Staphylococcus aureus*; *HIV,* human immunodeficiency virus; *CI*, confidence interval; *OR*, odds ratio
